# Differences in Molecular Responses to a Thermally Variable Preconditioning Treatment for Two Caribbean Coral Species

**DOI:** 10.1002/ece3.72108

**Published:** 2025-11-05

**Authors:** Allyson DeMerlis, Michael S. Studivan, Kevin Wong, Nash Soderberg, David Ehrens, Lys M. Isma, Katrina Rosing, Katrina Sophia Cocson, Rowan Thomas, Danielle Dvorkin, Patrick M. Kiel, Joseph D. Unsworth, Martine D'Alessandro, Ana M. Palacio‐Castro, Diego Lirman, Andrew C. Baker, Erinn M. Muller, Nikki Traylor‐Knowles, Ian C. Enochs

**Affiliations:** ^1^ University of Miami Cooperative Institute for Marine and Atmospheric Studies Miami Florida USA; ^2^ Ocean Chemistry and Ecosystems Division U.S. National Oceanic and Atmospheric Administration Atlantic Oceanographic and Meteorological Laboratory Miami Florida USA; ^3^ Marine Biology and Ecology University of Miami Rosenstiel School for Marine, Atmospheric, and Earth Science Miami Florida USA; ^4^ Mote Marine Laboratory Sarasota Florida USA

**Keywords:** coral, gene expression, preconditioning, thermal tolerance

## Abstract

Coral reefs around the world are increasingly threatened by rising ocean temperatures, leading to more frequent mass bleaching events. However, some corals, typically found in more thermally variable environments, have demonstrated resilience to thermal stress. Consequently, applying temperature variability for assisted acclimatization has been identified as a promising intervention for restoration efforts. While previous studies support this technique for thermal preconditioning, the underlying molecular mechanisms remain unclear. To address this research gap, we applied a variable temperature regime to promote preconditioning on two Caribbean coral species, the staghorn coral ( *Acropora cervicornis* ) and the knobby brain coral (*Pseudodiploria clivosa*) and evaluated changes in host and algal symbiont (Family Symbiodiniaceae) gene expression. Overall, the response to acclimatory treatments and the molecular mechanisms underlying them were species‐specific. *A. cervicornis* had a greater transcriptional response to the temperature treatment compared to *P. clivosa* (583 vs. 55 differentially expressed genes). In *A. cervicornis* , there was significant downregulation of key stress response genes, including peroxidases, nitric‐oxide synthase, and tumor necrosis factors, and an upregulation of genes involved in histone modifications. Importantly, these genes have been previously implicated in the generalized stress response of corals, suggesting that the molecular mechanisms of thermal preconditioning employ similar pathways. Considering the varying responses observed between species in this study, further research across a wider diversity of reef‐building coral species is necessary before implementation at the scale needed for restoration efforts.

## 1 Introduction

Ocean warming is one of the greatest threats to coral reefs worldwide (Hughes et al. 2017); therefore, understanding coral thermal tolerance and stress resilience is critically important for ecosystem preservation (Caruso et al. 2021). In Florida, active interventions to enhance coral resilience are of high priority, including selective breeding, assisted gene flow, holobiont community manipulations, and preconditioning (Bove et al. 2022; Cleves et al. 2020; Eakin et al. 2010; Putnam 2021; van Oppen et al. 2015, 2017). Preconditioning via exposure to a sublethal level of a stressor is particularly important for restoration efforts because it can induce more stress‐tolerant phenotypes of coral larvae and adults (Drury et al. 2022; Huffmyer et al. 2021; Majerova et al. 2021; van Oppen et al. 2015), and it has the potential to pass these benefits onto future generations (Liew et al. 2020). While mitigating anthropogenic carbon emissions is necessary to ensure the persistence of coral reef ecosystems, it is also imperative that scientists and marine managers investigate local approaches to delay the impacts of ocean warming on corals.

Current literature supports the application of thermal preconditioning to improve coral thermal tolerance in controlled laboratory experiments (Ainsworth et al. 2016; Barshis et al. 2013; Brown et al. 2024; Indergard et al. 2022; Majerova et al. 2021; Martell 2022). The majority of these studies, however, used static temperature treatments, which are unlikely to represent the natural conditions of reef environments. Temperature variability may also be an avenue for thermal preconditioning, as corals living in environments that naturally experience greater thermal variability tend to be more thermally tolerant (Ainsworth et al. 2016; Oliver and Palumbi 2011; Palumbi et al. 2014) and exhibit less bleaching during marine heatwaves (Donner 2011; Sully et al. 2019). This has been confirmed in some laboratory studies (Bay and Palumbi 2015; Bellantuono et al. 2012; DeMerlis et al. 2022; Dilworth et al. 2021; Drury et al. 2022; Mayfield et al. 2012), with a predominant focus on Pacific coral species and the photosynthetic response of the algal endosymbionts. Gene expression analysis has not yet been applied in the context of thermal preconditioning and would provide key insights into this process.

As several knowledge gaps remain regarding the molecular mechanisms of the coral bleaching response (Helgoe et al. 2024), investigating thermal preconditioning through a genetic lens is an important avenue for understanding coral resilience and acclimatization. The current literature has highlighted key genes that are commonly activated following heat stress exposure, including heat‐shock proteins (HSPs), tumor necrosis factor (TNF) receptors, and other genes involved in cell death signaling, the innate immune response, and the oxidative stress response (Barshis et al. 2013; Bay and Palumbi 2015; Bellantuono et al. 2012; Drury et al. 2022; Majerova et al. 2021; Mayfield et al. 2012). However, the duration of thermal stress dictates the magnitude of the transcriptional response, with short‐term treatments leading to a reduction in gene expression (Bay and Palumbi 2015) and long‐term exposure being associated with constitutively higher baseline expression of stress‐response genes, also referred to as “gene front‐loading” (Barshis et al. 2013; Palumbi et al. 2014). Additionally, a meta‐analysis of transcriptomic datasets found that corals in the genus *Acropora* employ two distinct gene expression profiles based on the level of heat stress applied; the first corresponded with high levels of various stressors and was strongly correlated across experiments, and the second corresponded with low levels of stress and had much greater variation (Dixon et al. 2020). These studies demonstrate that further research is necessary to contextualize the genetic pathways employed during thermal preconditioning in the coral host and its algal symbionts.

The molecular mechanisms of temperature variability for thermal preconditioning are understudied in Caribbean corals, particularly for restoration priority species. To address these gaps, we applied a 28‐day variable temperature treatment to enhance the thermal tolerance of two Caribbean coral species with different life history strategies and algal endosymbiont community associations. We then measured changes in coral host and symbiont gene expression patterns before and after the treatment to identify the underlying molecular mechanisms employed by each species. To evaluate changes in thermal tolerance due to the variable temperature treatment alone, a rapid heat‐stress assay was employed immediately following the ex‐situ experimental treatment. The species assessed, *Acropora cervicornis* and *Pseudodiploria clivosa*, are commonly propagated and outplanted to promote coral reef restoration across Florida and are thus important to investigate for improving the efficacy of assisted acclimatization and understanding their mechanisms for thermal tolerance.

## 2 Materials and Methods

### 2.1 Coral Collection and Acclimation

In February 2022, three *A. cervicornis* genotypes were collected from the University of Miami in‐situ coral nursery off Key Biscayne, FL (25.6763, −80.0987, ~8 m depth), and three colonies of *P. clivosa* were opportunistically sourced from a seawall construction project in the Port of Miami, FL (25.7705, −80.1524, ~3 m depth). The genotypes of *A. cervicornis* were previously confirmed as distinct using single nucleotide polymorphism genotyping (Kitchen et al. 2019), whereas the *P. clivosa* colonies were not genotyped (Supporting Information, Table S1). For clarity, “genotypes” and “colonies” will be henceforth referred to as “colonies.”

All colonies were brought to the Experimental Reef Laboratory at the University of Miami's Cooperative Institute for Marine and Atmospheric Studies and fragmented into 5 cm‐long fragments (for *A. cervicornis* ) and 5 cm^2^ fragments (for *P. clivosa*) using a Griffin diamond bandsaw (*N* = 146–149 fragments per species). In the case of *A. cervicornis* , apical tips and additional branches were removed from all fragments to constrain variability in growth and calcification rates. Fragments were acclimated to the ex‐situ environment for 30 days at 24°C, mimicking in‐situ temperatures at the time of collection. Temperatures were then increased gradually at a rate of 0.5°C day^−1^ to a temperature of 28°C. The setpoint of 28°C was chosen as the baseline temperature for the experiment because it represents an average in‐situ temperature for the Miami region and has not been shown to cause visible stress to corals collected in Miami (DeMerlis et al. 2022; Enochs et al. 2023; Palacio‐Castro et al. 2023). Flow rates and diurnal setpoints are detailed in Supporting Information S1.

### 2.2 Temperature Variability Treatment

**FIGURE 1:**
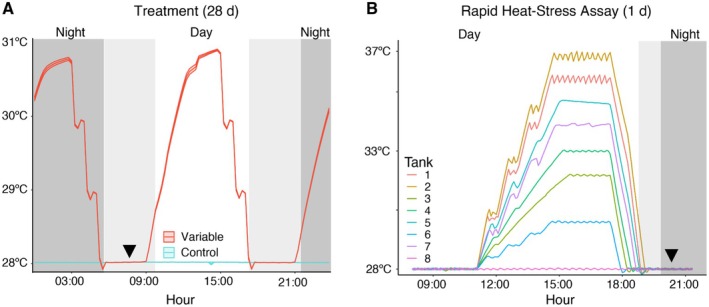
(A) Temperature profiles for the variable temperature treatment. Mean tank temperature (*N* = 4 tanks per treatment) every 15 min over 28 days for the variable (red) and control (blue) groups. Ribbons represent standard error of the mean temperature. Gray shading represents change in light levels across a diel cycle. The triangle denotes the time of day that physiological metrics were taken. (B) Rapid heat‐stress assay temperatures, where each tank was randomly assigned to a temperature between 28°C and 37°C (*N* = 8). Thirty minutes after sundown, photosynthetic efficiency of each coral was measured (denoted with black triangle).

Coral fragments of both species were randomly assigned to one of eight 90 L glass aquaria (0.58 × 0.58 × 0.27 m), and fragments from each colony were evenly distributed to control for any potential tank effects. For the variable temperature treatment, twice per day for 28 days in four of the tanks, temperatures increased to 31°C over 3 h, then were held at 31°C for 3 h, and subsequently decreased to 28°C (Figure 1A). The maximum setpoint of 31°C was selected as a high temperature for sublethal stress exposure based on previous preconditioning studies (i.e., Dilworth et al. 2021; DeMerlis et al. 2022), as well as in‐situ summertime temperatures from the Miami and Biscayne Bay regions from 2018 to 2023 (Enochs et al. 2023; Palacio‐Castro et al. 2023). This variable profile also replicated the methodology described in DeMerlis et al. (2022), where a fluctuation of 3°C per day occurred both during the day and night periods to maximize sublethal exposure to thermal stress while still providing recovery periods throughout the treatment. The remaining four tanks were assigned as the control treatment and were kept at a static temperature of 28°C for 28 days. Temperatures were controlled with aquarium heaters and titanium chiller coils and logged every 5 min using custom software written in LabVIEW (National Instruments) as previously described (Enochs et al. 2018). Experimental tank conditions and the coral feeding regime are described in Supporting Information S1.

### 2.3 Rapid Heat‐Stress Assay

One day after the variable temperature treatment ended, a rapid heat‐stress assay was used to evaluate whether prior exposure to temperature variability influenced coral thermotolerance. The rapid heat‐stress assay involved an elevated temperature exposure over 3 h, followed by a 3 h hold at the maximum temperature (eight temperature levels, one for each tank, ranging from 28°C to 37°C), and then a decrease back to the ambient temperature (28°C) over 1 h (Figure 1B). The temperatures were selected based on prior studies that applied a rapid heat‐stress assay, the Coral Bleaching Automated Stress System (CBASS), with *A. cervicornis* (Cunning et al. 2021, 2024; Voolstra et al. 2020). The decrease in temperature coincided with sunset so that dark acclimation occurred and was followed by measurements of algal endosymbiont photochemical efficiency.

### 2.4 Coral‐Algal Physiology

Photosynthetic efficiency, measured from the dark‐adapted yield of photosystem II (*F*_V_/*F*_M_), provides a metric to assess health and functioning of the coral's dinoflagellate endosymbionts and has become a proxy for coral holobiont thermal tolerance (Alderdice et al. 2022; Caroselli et al. 2015; Nielsen et al. 2022; Ralph et al. 2016; Voolstra et al. 2020; Warner et al. 1996, 1999). To measure *F*_V_/*F*_M_, corals were first dark‐acclimated for 30 min and then measured using the imaging pulse amplitude‐modulated fluorometer (Imaging‐PAM MAXI Version, Walz, Germany). One area of interest was selected in the center of each coral fragment for measurements. Software settings were customized to include the following parameters in each session: measuring light intensity = 1, measuring light frequency = 1, damping = 2, saturating pulse intensity = 7, and saturating pulse width = 4. The gain setting was adjusted as necessary to produce an F_T_ measurement above 0.12. Data was exported as CSV files and read into R for analysis using code adapted from the custom script “IPAM2R” (Cunning 2017; DeMerlis 2023a).

To assess the influence of the variable temperature treatment on the thermotolerance of *A. cervicornis* and *P. clivosa*, algal endosymbiont photosynthetic efficiency was measured at Day 0 and Day 28 of the variable temperature treatment, and at the end of the rapid heat‐stress assay. To assess the effect of the variable temperature regime on photosynthetic efficiency, Welch's ANOVA was performed on pre‐treatment‐normalized *F*_V_/*F*_M_ values for each species separately, as variances were unequal, but data was normally distributed. To assess treatment differences in the mean *F*_V_/*F*_M_ for each species following the rapid heat‐stress assay, one‐way ANOVAs were performed at each rapid heat‐stress temperature applied. Data met the assumptions of normality (Shapiro–Wilk test) and heteroscedasticity (Levene's test). Additionally, coral calcification and changes in tissue coloration were measured over the course of the variable temperature treatment. Details of methodology and statistical analysis can be found in the Supporting Information S1.

### 2.5 Tag‐Seq Library Preparation, Sequencing, and Bioinformatics

To assess the effect of the variable temperature treatment on coral host and algal endosymbiont gene expression, small tissue samples (~1 cm^2^) were obtained from randomly selected coral fragments at Day 0 and Day 28 of the treatment, immediately preserved in DNA/RNA Shield (Zymo Research, Cat #R1100‐250), and frozen at −80°C until processing. Different coral fragments were sampled at each time point to avoid a potential confounding effect of repeated sampling. Four fragments of each species, colony, and treatment were collected across treatment tanks (*N* = 48 total for each species). To ensure consistency across time points, corals were sampled during peak daytime settings and only when the variable temperature‐treated coral temperature tanks were at 28°C, as this was the temperature of the control tanks.

Total RNA was extracted following the DNA/RNA Biomics Miniprep extraction protocol (Zymo Research, Cat #R2002), including the optional HRC Inhibitor removal step and RNA Clean and Concentrator‐5 kits (Zymo Research, Cat #R1014). All samples were normalized to 10 ng μL^−1^ and sent to the University of Texas at Austin Genome Sequencing and Analysis Facility for library preparation and sequencing. Tag‐Seq library preparation was utilized (Meyer et al. 2011), and libraries were pooled and sequenced on an Illumina NovaSeq S2 SR100.

Raw sequences were processed using code adapted from custom Perl scripts (Matz 2015b; Studivan 2020). Sequences were deduplicated and adaptor‐trimmed using 64‐fold degenerate 5′‐headers and the first 30 bases of the read sequence, then filtered for quality with a trimming threshold of 15 using cutadapt v4.4 (Martin 2011). Sequences were then mapped using Bowtie2 v2.5.2 (Langmead and Salzberg 2012) simultaneously to the respective host genome or transcriptome— *A. cervicornis* (Locatelli et al. 2024), *P. clivosa* (Avila‐Magaña et al. 2021)—and a reference containing Symbiodiniaceae 28S sequences across the four main genera—*Symbiodinium* spp. and *Durusdinium* spp. (Shoguchi et al. 2021), *Breviolum* spp. (Avila‐Magaña et al. 2021), and *Cladocopium* spp. (Davies et al. 2018). Sequences which aligned to both host and symbiont genomes or transcriptomes were discarded.

Samples from *A. cervicornis* had 100% alignment to *Symbiodinium* spp. and *P. clivosa* samples had 98.9% alignment to *Breviolum* spp.; therefore, the respective symbiont genomes were concatenated to the host genome or transcriptome and used for reference alignment. The program SAMtools v1.3 (Danecek et al. 2021) was used to quantify gene counts. Lastly, genome and transcriptome annotations for *A. cervicornis* and *P. clivosa* were created using protocols in the GitHub repositories “annotatingTranscriptomes” (Matz 2015a) and “emapper_to_GOMWU_KOGMWU” (Matz 2018), which employ *eggnog‐Mapper* (Huerta‐Cepas et al. 2017) to generate predicted gene names based on consensus orthologs, as well as match Gene Ontology (GO) and euKaryotic Orthologous Group (KOG) functions.

Following deduplication, the mean number of reads (± standard error of the mean) across all samples for *A. cervicornis* was 11.8 ± 0.4 million reads, and 9.8 ± 1.0 million reads for *P. clivosa*. Following trimming, this was reduced to 3.6 ± 0.2 million reads for *A. cervicornis* and 0.7 ± 0.08 million reads for *P. clivosa*. For *A. cervicornis* , the alignment rate was 61% ± 0.6%, which resulted in coverage of 30,122 host and 43,816 symbiont genes. For *P. clivosa*, the alignment rate was 91.7% ± 0.2%, which resulted in coverage of 59,947 host and 26,253 symbiont genes. Differences between species are likely due to differences in RNA extraction yield, sample quality, and reference genome/transcriptome quality. The counts per sample and alignment rates can be found within this study's GitHub repository (DeMerlis 2023b).

### 2.6 Differential Gene Expression Analysis

Read counts for both species were imported into R and analyzed for differential gene expression using the *DESeq2* R package (Love et al. 2014). Each species' host and symbiont differential gene expression analysis were conducted separately. The analysis for *Symbiodinium* reflects only samples from *A. cervicornis* , and the analysis for *Breviolum* reflects only samples from *P. clivosa*. Genes with < 10 counts across all samples were removed, and the *DESeq2* model was created with the design: “~Colony + Treatment”. Data was then transformed using a variance stabilizing transformation (Love et al. 2014). Outlier detection was run on transformed data using the *arrayQualityMetrics* R package (Kauffmann et al. 2009), and outliers were removed from each dataset based on the default sample array distance criterion.

To account for the samples collected prior to the start of the variable temperature treatment compared to the end‐of‐treatment samples, three groups were assigned as: “Initial” (Day 0), “Control” (Day 28), and “Variable” (Day 28). The *DESeq2* model was transformed using a variance‐stabilized transformation (Love et al. 2014) and then was visualized via a principal coordinates analysis (PCoA), applying Manhattan distances. Next, a permutational multivariate analysis (PERMANOVA) was employed on the Manhattan distances determined from principal coordinates to test significance of colony and treatment as fixed effects using 1e^6^ permutations and the “*adonis2()*” function from the *vegan* R package (Oksanen 2016).

The Wald test was used to determine the number of differentially upregulated and downregulated genes per contrast (“Control vs. Initial,” “Variable vs. Initial,” and “Variable vs. Control”), with significantly differentially expressed genes (DEGs) defined by a false discovery rate (FDR)‐adjusted *p*‐value (*p*‐adj) cut‐off of 0.05 and a log‐2‐fold change (L2FC) cut‐off of 1 (upregulated) and −1 (downregulated). Venn diagrams of common and unique DEGs between the three treatment comparisons were generated using the *ggvenn* R package (Yan and Yan 2023).

### 2.7 Functional Enrichment Analysis

To assess whether functional groups of genes were enriched in each treatment comparison and between species, GO enrichment analysis was conducted using Mann–Whitney U tests in the R packages *GO_MWU* (Wright et al. 2015) based on rankings of the *DESeq2*‐derived log *p*‐value (lpv) and L2FC. Each *DESeq2* contrast was analyzed independently: “Control vs. Initial,” “Variable vs. Initial,” and “Variable vs. Control.” The GO enrichment terms are organized into the following three divisions to explain functionality of gene groups: Biological Process (BP), Molecular Function (MF), and Cellular Component (CC). For the rank‐based enrichment analysis of each contrast, GO terms were filtered to contain at least five genes and < 10% of the total number of genes, and the cluster cut height threshold was set to 0.25 for merging similar GO terms. All functional enrichment analyses were tested on all expressed genes for significance at the alpha level of 0.05 based on the FDR‐adjusted *p*‐value. For ease of visualization, bubble plots were generated with the R package *ggplot2* for only the top five significant GO terms for each division (BP, MF, CC), which were derived using L2FC‐ranked enrichment for “Variable vs. Control” and “Variable vs. Initial,” with significant “Control vs. Initial” GO terms removed first.

A transcriptomic meta‐analysis of the genus *Acropora* found that the severity of thermal stress experienced influenced the outcome of gene expression and characterized two distinct genetic environmental stress responses (ESRs): a type “A” response for high levels of various stressors and a strong correlation in gene expression patterns across experiments, and a type “B” response that was more variable across experiments which applied low levels of stress (Dixon et al. 2020). The meta‐analysis produced lists of GO BP terms based on rankings of the *DESeq2*‐derived log‐transformed *p*‐value (lpv). Subsequently, comparisons of the type “A” and type “B” ESRs have been applied in recent studies to evaluate whether genetic outcomes following thermal challenges are consistent with the findings of the 2020 meta‐analysis (Aichelman et al. 2024; Wuitchik et al. 2024). Following this methodology, the GO BP lpv‐ranked enrichment terms from this study, specifically for the “Variable vs. Control” contrast, were used to calculate rank‐based correlations for *A. cervicornis* to the *Acropora* meta‐analysis (Dixon et al. 2020). The goal was to assess whether the temperature variability treatment of *A. cervicornis* in this study correlated with the type “A” or type “B” ESR. The GO‐MWU enrichment analysis provided delta‐ranks for each GO term, which is a relative value within the experimental dataset to relate the expression of a group of genes associated with a given GO term between treated and control samples (Wright et al. 2015). GO BP terms shared between this study and the *Acropora* meta‐analysis were plotted using the delta‐ranks, with the type “A” and type “B” GO terms separated based on the meta‐analysis (Dixon et al. 2020). The number of GO BP terms shared between this study and the *Acropora* meta‐analysis were encoded as a heatmap on the correlation graphs, and lines of best fit were plotted to demonstrate the positive or negative correlation with the type “A” or type “B” ESR.

## 3 Results

### 3.1 Species‐Specific Physiological Responses to Variable Temperature Treatment and Heat Stress

**FIGURE 2:**
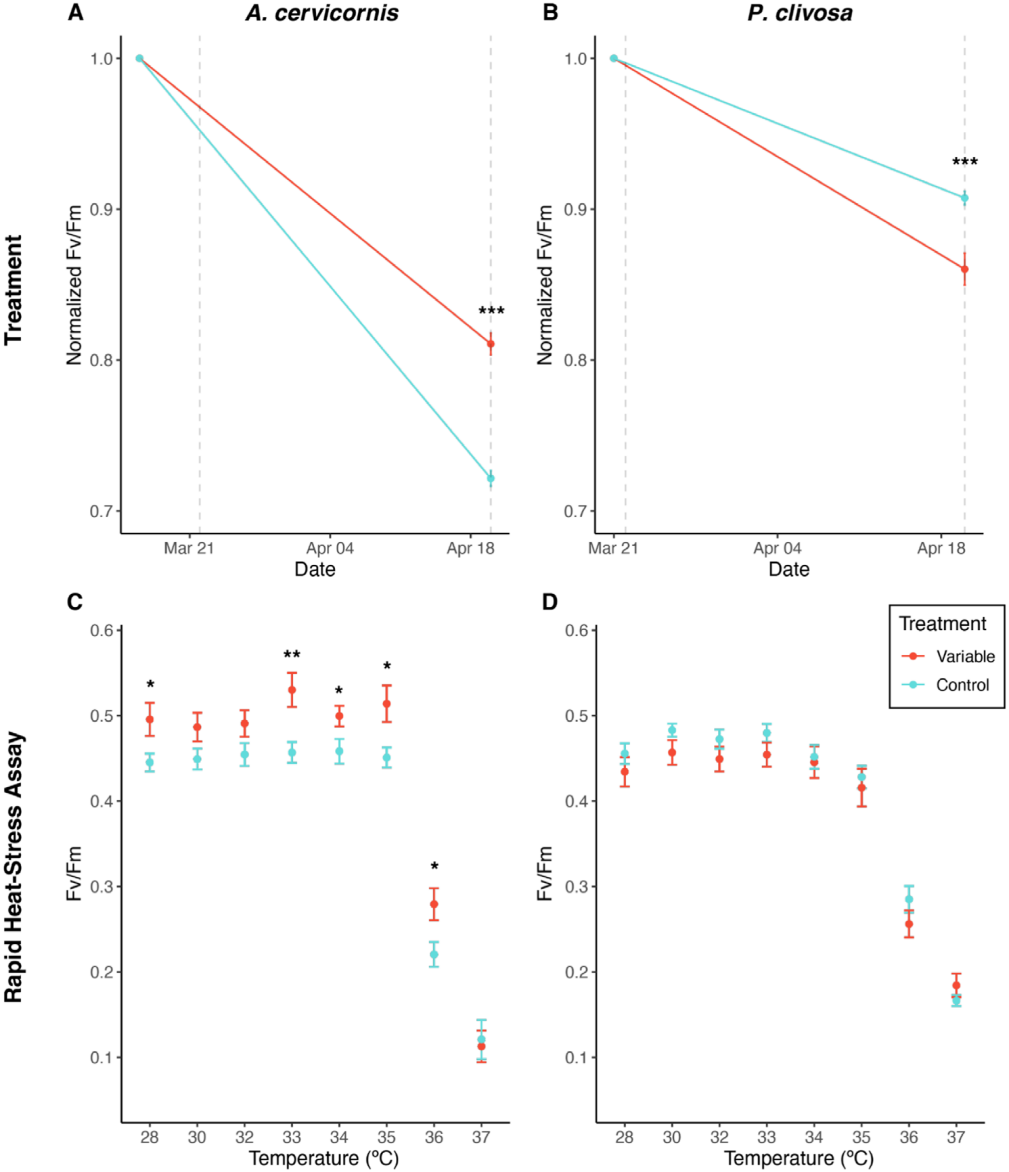
Photosynthetic efficiency, or *F*_V_/*F*_M_, of the algal endosymbionts following the variable temperature treatment (A, B) and rapid heat‐stress assay (C, D). Vertical dashed lines indicate start and end of variable temperature treatment. Values plotted are the mean (± standard error of the mean). ANOVA results from significance between treatments are denoted with asterisk, significance codes *p < 0.05, **p < 0.01, and ***p < 0.001 (Tables S5–S7 in Supporting Information S1).

The variable temperature treatment had significant effects on the photosynthetic efficiency of the algal endosymbionts of both *A. cervicornis* and *P. clivosa*; however, trends were the opposite for each species (Welch's ANOVA, *p* < 0.001). For *A. cervicornis* , the control group had significantly greater declines in photosynthetic efficiency, while for *P. clivosa*, the variable temperature‐treated corals experienced significantly greater declines (Figure 2A,B). Corals in ex‐situ conditions have previously shown declines in photosynthetic efficiency, even in the control group (i.e., Dilworth et al. 2021; DeMerlis et al. 2022), which may be due to differences in light and food availability influencing coral‐algal physiology. In the present study, for *A. cervicornis* , the treatment had a similar influence on coral tissue coloration, where variable temperature‐treated corals maintained significantly higher color scores (Kruskal‐Wallis test, *p* < 0.001, Figure S2A, Supporting Information S1). However, there was no significant difference in coral tissue coloration between control and variable temperature‐treated *P. clivosa* (Figure S2B, Supporting Information S1). Coral fragments of both species were healthy throughout the experimental treatment period, demonstrated by increased calcification over 28 days (Figure S1, Supporting Information S1) and maintenance of coral tissue coloration (Figure S3, Supporting Information S1). There was no significant impact of the treatment on calcification for either species (Figure S1, Supporting Information S1).

The rapid heat‐stress assay revealed species‐specific differences in thermal tolerance following the variable temperature treatment. For *A. cervicornis*, variable temperature‐treated corals had significantly higher photosynthetic efficiencies at 33 to 36°C compared to controls (ANOVA, *p* < 0.05, Figure 2C). For *P. clivosa*, however, there were no significant differences in photosynthetic efficiency between control and variable temperature‐treated corals at any of the rapid heat‐stress assay temperatures (Figure 2D).

### 3.2 Greater Effect of Treatment on Host and Symbiont Gene Expression Patterns for *A. cervicornis* Compared to *P. clivosa*

**FIGURE 3:**
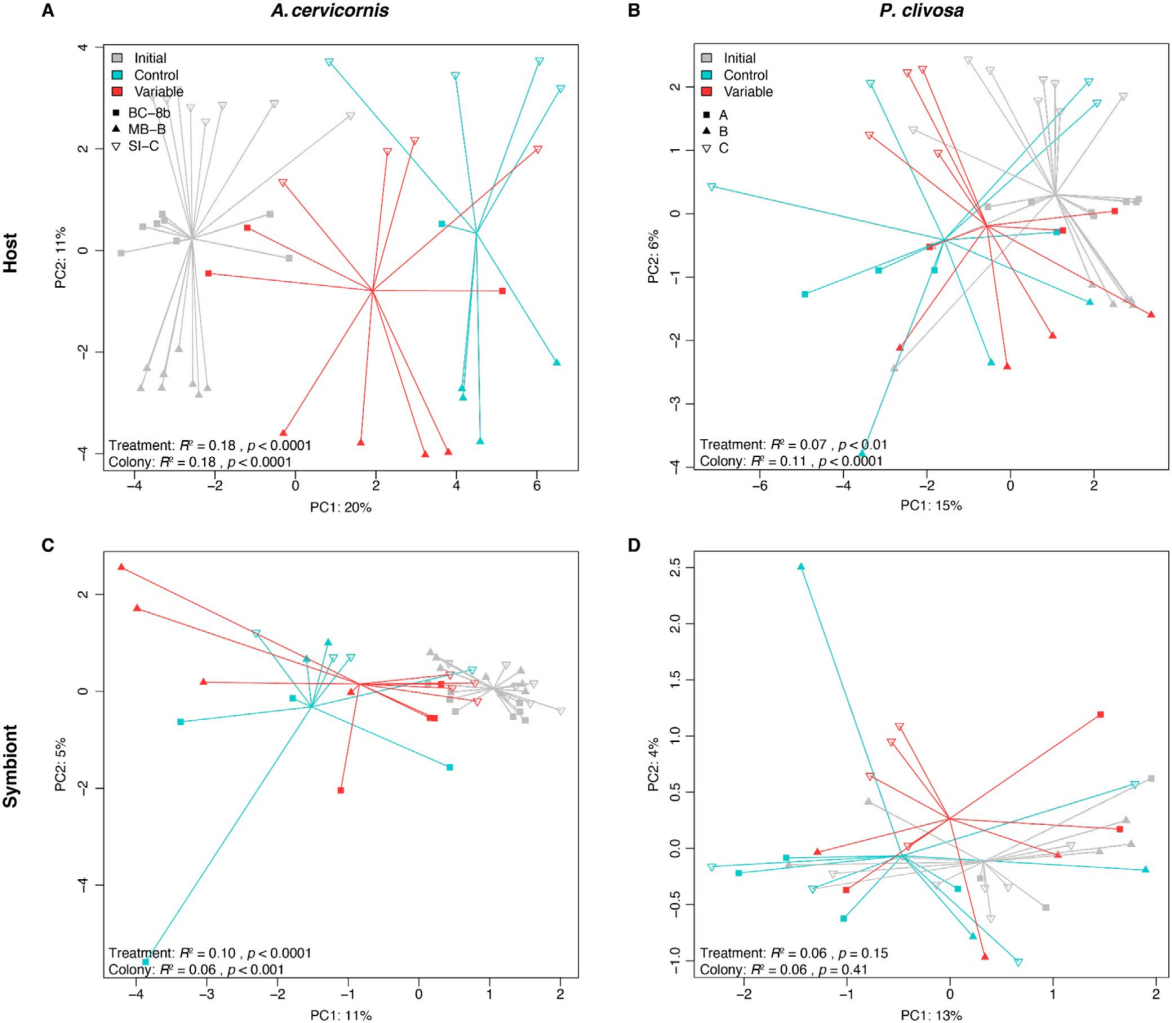
Principal coordinates analysis (PCoA) for each coral species (A, B) and their associated symbionts (C, D), demonstrating sample variation based on treatment (color) and colony (shape). Colony names for *A. cervicornis* were derived from the local genotype name assigned in the in‐situ coral nursery, which correspond to original region of collection from wild thickets (Broward County (BC)‐8b, Miami Beach (MB)‐B, and Sunny Isles (SI)‐C). The STAGdb Clonal IDs for these genotypes of *A. cervicornis* are listed in Table S1 in Supporting Information S1. Colony names for *P. clivosa* were arbitrarily assigned as “A”, “B”, and “C”. Test statistics are from PERMANOVA models (Table S8 in Supporting Information S1).

The temperature treatment had significant effects on host gene expression patterns for both *A. cervicornis* (PERMANOVA, *p* < 0.001) and *P. clivosa* (PERMANOVA, *p* < 0.01), however, PCoA ordination demonstrated a greater distinction between treatment groups for *A. cervicornis* compared to *P. clivosa* (Figure 3A,B). This relationship was maintained for each symbiont species, whereby *Symbiodinium* spp. was significantly influenced by treatment (PERMANOVA, *p* < 0.001) but *Breviolum* spp. was not (Figure 3C,D). Following *DESeq2*, pairwise differential expression tests revealed that the greatest number of differentially expressed genes (DEGs) between variable temperature‐treated and control corals were observed for *A. cervicornis* (200 upregulated, 207 downregulated), followed by *P. clivosa* (3 upregulated, 7 downregulated), and *Symbiodinium* spp. (2 downregulated) (Table S9 in Supporting Information S1). *Breviolum* spp. had no significant DEGs for the variable versus control corals.

**FIGURE 4:**
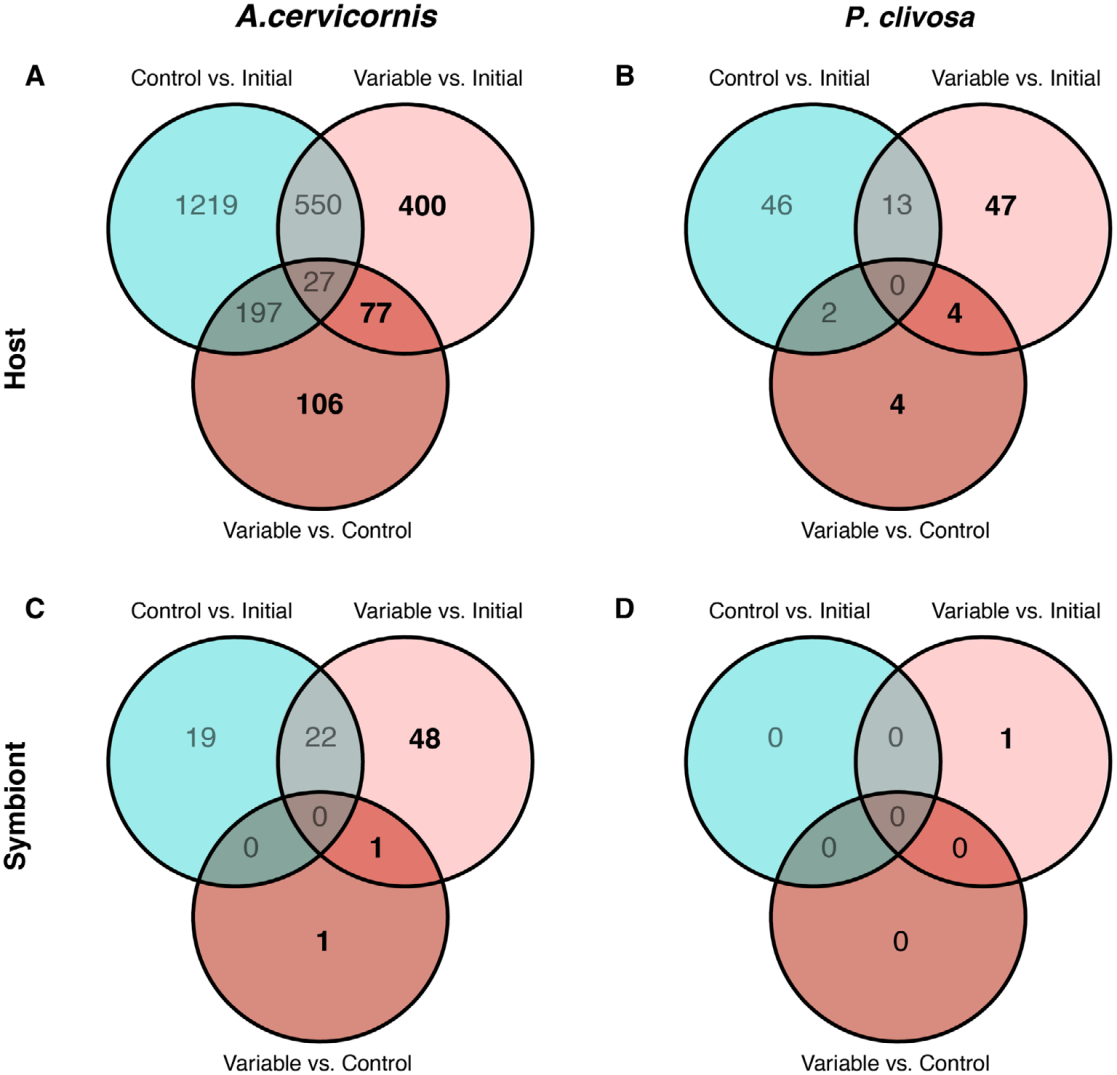
Venn diagrams of significant differentially expressed genes for each coral species (A, B) and their respective algal endosymbionts (C, D) based on each treatment comparison and filtered based on a criterion of *p*‐adj < 0.05 and |L2FC| > 1. Genes of interest for implications of the variable temperature regime are bolded.

Within each species, the greatest number of DEGs came from *DESeq2* pairwise comparisons with the initial timepoint, indicating a temporal shift in gene expression after 28 days. To determine which genes were specifically driving the response to thermal variability, DEGs from the pairwise *DESeq2* control versus initial timepoint were removed from the significant DEG lists for the two pairwise comparisons which encompassed the variable temperature treatment: variable temperature‐treated versus initial and variable versus control. This resulted in a combination of the DEGs from the two pairwise comparisons, totaling 583 DEGs for *A. cervicornis* , 55 DEGs for *P. clivosa*, 50 DEGs for *Symbiodinium* spp., and 1 DEG for *Breviolum* spp. (Figure 4).

When looking at annotated genes with high absolute L2FC values, different trends emerged for each species. For variable temperature‐treated *A. cervicornis* , several HSPs were significantly downregulated (Table S2). There was significant downregulation of genes involved in oxidative detoxification, such as nitric oxide synthase, cytochrome P450, and several peroxidases. Additionally, there was downregulation of genes involved in pathogen recognition, including a TNF receptor‐associated factor, C‐type lectin, and NF‐kappaB‐inducing kinase activity. There was differential regulation in variable temperature‐treated *A. cervicornis* for metabolic genes, including upregulation of cAMP‐dependent protein kinase regulator activity and downregulation of calcium ion binding and several hydrolases. Lastly, there was differential regulation in transcription and gene accessibility, including upregulation of histone H2A and downregulation of WD40 repeats, helicase activity, and chromatin remodeling (Table S2).

For *P. clivosa*, there was significant upregulation of metabolic genes in variable temperature‐treated corals, including an ATPase, a hydrolase, zinc ion binding, and oxidoreductase activity. Additionally, there was significant downregulation of stress response genes, including an HSP and protein folding activity (Table S4).

Variable temperature‐treated *Symbiodinium* spp. hosted within *A. cervicornis* demonstrated patterns of significant upregulation of cytochrome enzymes involved in the electron transport chain, including cytochrome c oxidase and a component of the cytochrome b6‐f complex. Additionally, there was upregulation of antioxidant activity and cellular metabolism, including hydrolase, peptidase, and oxidoreductase activity (Table S3). Lastly, for *Breviolum* spp., only one differentially expressed gene was annotated, which was a metabolic enzyme that was significantly upregulated in variable temperature‐treated corals (Table S5).

### 3.3 GO Enrichment Revealed Treatment‐Specific Heat Stress and Immune Response in *A. cervicornis*

**FIGURE 5:**
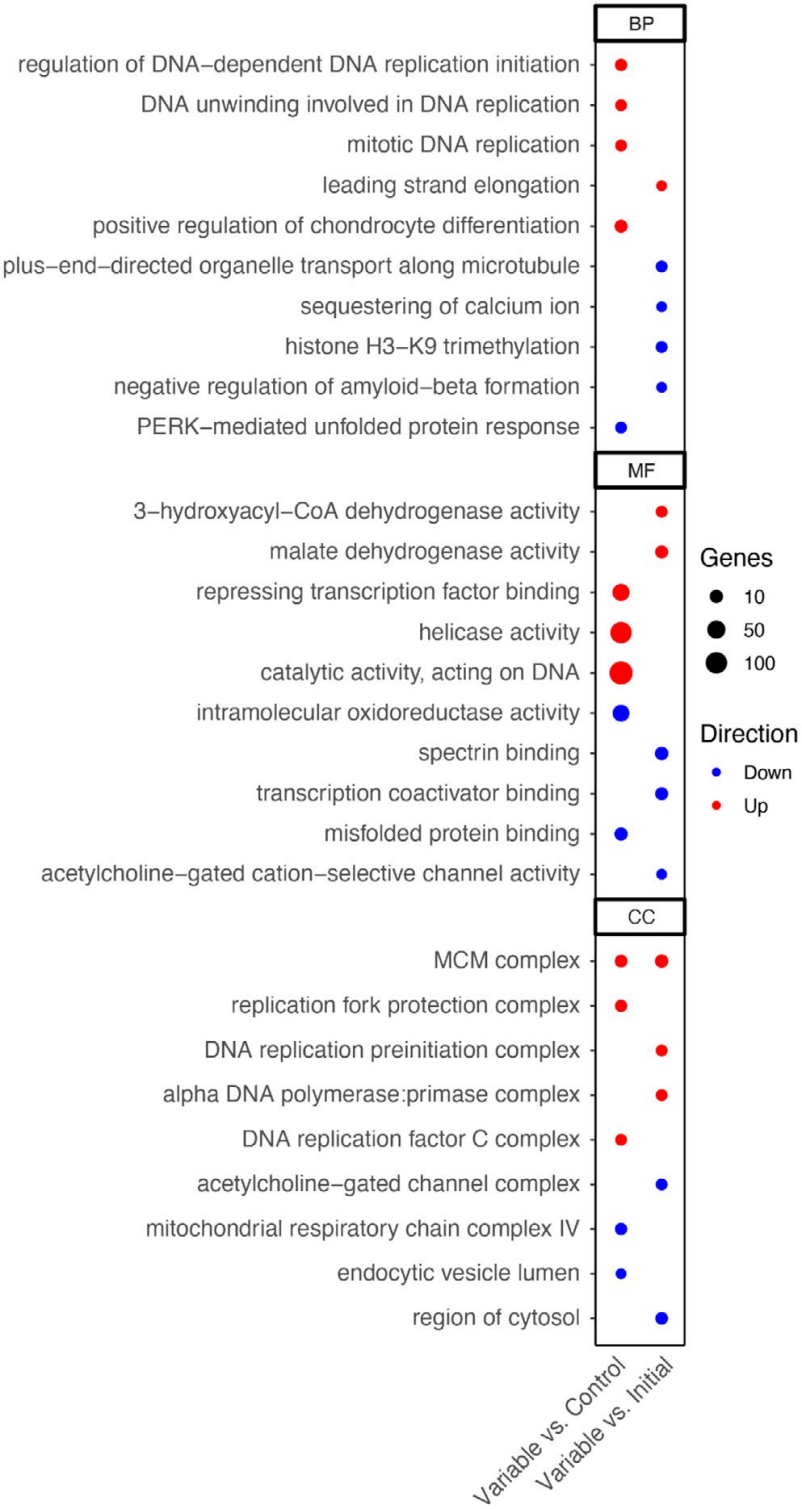
Bubble plots of top significantly differentially expressed gene ontology (GO) terms for *Acervicornis cervicornis* host variable temperature‐treated contrasts, which were determined using delta‐rank values comparing *DESeq2*‐derived log‐2‐fold changes (L2FC). The size of the bubble represents the number of genes and color represents the overall direction of enrichment for the GO term. GO terms are separated into three categories: Biological Process (BP), Molecular Function (MF), and Cellular Component (CC).

Only *A. cervicornis* had a sufficient number of significant DEGs to produce significant GO terms for the method of GO enrichment analysis used in this study, resulting in 210 GO terms across three categories for the variable temperature‐treated versus control corals (BP: 160, MF: 15, CC: 89) and 1075 GO terms for the variable temperature‐treated versus the initial timepoint (BP: 802, MF: 117, CC: 156; Table S10 in Supporting Information S1). After filtering out GO terms which also overlapped with the control versus initial timepoint corals, several significant trends emerged, including downregulation of the heat stress response and immune response. One GO term was specifically related to heat response (BP, GO:0009408), while related terms, such as the unfolded protein response (BP, GO:0036499) and misfolded protein binding (MF, GO:0051787), were significantly downregulated in variable temperature‐treated corals (Figure 5). There were also several immune‐related GO terms which were significantly downregulated in variable temperature‐treated *A. cervicornis* , including phagocytosis (BP, GO:0006909), apoptotic cell clearance (BP, GO:0043277), regulation of defense responses to bacteria and viruses (BP, GO:0050691, GO:1900424), regulation of NF‐kappaB transcription factor activity (BP, GO:0032088), and responses to TNFs (BP, GO:0034612, GO:0071356) (Table S6). Lastly, several GO terms related to epigenetic processes were also significantly downregulated, including histone H3‐K9 modifications (BP, GO:0036124, GO:0051567, GO:0061647) and epigenetic regulation of gene expression (BP, GO:0006342, GO:0045814, GO:0097549, GO:0034401) (Table S6).

**FIGURE 6:**
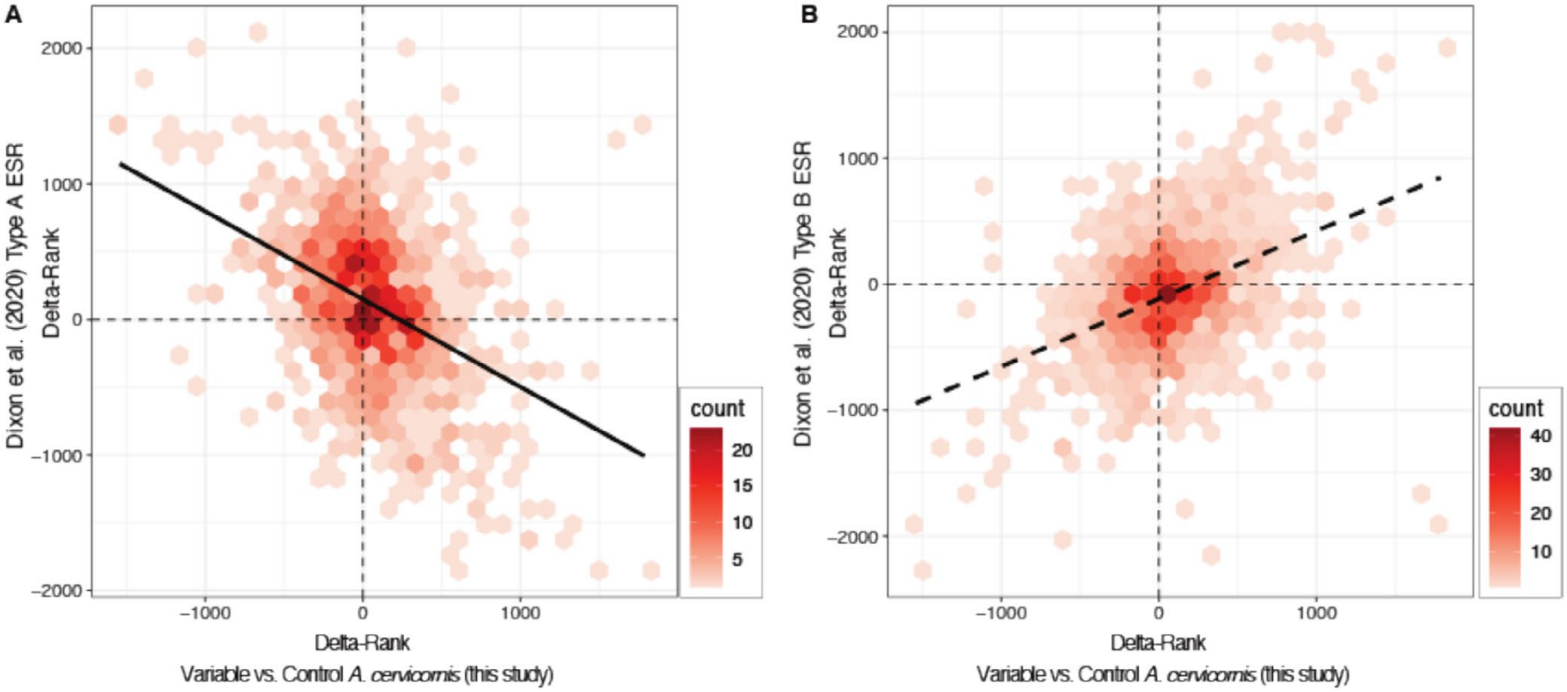
Comparison of delta‐ranks of gene ontology (GO) enrichment terms under Biological Process (BP) between the variable temperature‐treated versus control *Acervicornis cervicornis* from this study and the two types of Environmental Stress Response (ESR) characterized from a meta‐analysis of the genus *Acropora* in Dixon et al. (2020). The count heatmap encodes the number of shared GO BP terms between the two studies, and their distribution along the line of best fit reflects a greater positive or negative correlation with either type “A” (A) or type “B” (B) ESR. A negative slope suggests that the genetic responses in this study (based on GO BP terms) and the meta‐analysis ESR type are more dissimilar, while the positive slope suggests functional similarities in response to a thermal challenge.

When taking all the enriched GO terms from the variable temperature‐treated *A. cervicornis* in comparison with the control group in this study and comparing them with the GO terms from the Dixon et al. (2020) *Acropora* meta‐analysis, there was a significant negative correlation with the type “A” ESR and a significant positive correlation with the type “B” ESR (Figure 6). Many GO terms which were upregulated in the type “A” ESR but were downregulated in variable temperature‐treated *A. cervicornis* were related to the immune response, including antigen processing and presentation (GO:0002474), defense response to virus (GO:0051607), and phagocytosis (GO:0006911, GO:0050764) (Table S7). Conversely, significantly upregulated GO terms in both the variable temperature‐treated *A. cervicornis* and the type “B” ESR were related to DNA replication, including DNA strand elongation (GO:0006271, GO:0022616) and cell cycle DNA replication (GO:0044786) (Table S8).

## 4 Discussion

In this study, we found that the same mechanisms underpinning the thermal stress response are similarly employed in preconditioning, yet the direction of differentially expressed genes contrasts the findings in previous studies, in particular the hypothesis of constitutive “front‐loading” as a means of acclimatization (Barshis et al. 2013). The equivocal transcriptional responses of acroporids documented in current literature may be due to the level and duration of thermal stress applied. For example, Bay and Palumbi (2015) found no significant differences in gene expression between stable versus variable temperature treatments with *A. nana* following the treatment itself but found a reduction in gene expression levels in variable‐temperature acclimated corals following heat stress. This “muted” stress response in pre‐acclimated corals contrasts with work applying long‐term variable temperature acclimatization in *A. hyacinthus* , with shifts in baseline expression seen over a year‐long period (Barshis et al. 2013; Palumbi et al. 2014). Thus, applying both short‐ and long‐term variable temperature treatments for a variety of coral species will be important for the efficacy of thermal preconditioning, as was recently demonstrated (Brown et al. 2024).

An important confounding variable in this study which could not be experimentally tested was the role of source location for the *A. cervicornis* and *P. clivosa* colonies collected for this experiment. Notably, the species were collected from different locations and different depths. The colonies of *A. cervicornis* were originally collected from three different reefs in the northern Florida Reef Tract off Miami, FL, and have been propagated and maintained in an in‐situ coral nursery near Emerald Reef, at a depth of 8 m, off Key Biscayne, FL for greater than 2 years. The colonies of *P. clivosa* were collected off a shallow seawall at a depth of 3 m within the Port of Miami. The in‐situ nursery and the Port of Miami experience differences in temperature, seawater pH, salinity, and nutrient concentrations (Enochs et al. 2023). These environmental parameters, in tandem with the differing depths and thus light irradiance exposure, likely have a significant role in shaping the coral's response to the variable temperature preconditioning treatment. While previous work has identified significant differences in gene expression between reef‐native and Port of Miami‐native corals (Rubin et al. 2021), this study cannot parse apart differences in species‐specific responses versus environmental preconditioning in *A. cervicornis* and *P. clivosa*.

### 4.1 Dose of Thermal Variability May Benefit the Photophysiology of Certain Coral‐Algal Associations Over Others

The present study investigated the molecular mechanisms following variable temperature preconditioning in threatened Caribbean coral species that are propagated as part of coral reef restoration efforts. Previously, the thermal tolerance of acroporids has been successfully enhanced by temperature variability observed as delayed bleaching, reduced bleaching severity, and/or a reduction in photophysiological damage (Brown et al. 2024; DeMerlis et al. 2022; Palumbi et al. 2014; Thomas et al. 2018). This study demonstrated that variable temperature‐treated *A. cervicornis* maintained a higher photosynthetic efficiency following thermal stress; however, the treatment had the opposite effect on *P. clivosa*, whereby untreated controls experienced less photophysiological decline. The level of temperature variability applied in this study was replicated based on previous work using *A. cervicornis* (DeMerlis et al. 2022); however, the amplitude required to observe a benefit in *P. clivosa* may be different. This may be driven by their different life‐history strategies, as *A. cervicornis* is a fast‐growing, competitive species while *P. clivosa* is slow‐growing and considered to be more stress‐tolerant (Darling et al. 2012), and is commonly found in nearshore, more thermally variable environments (van Woesik et al. 2020). Brown et al. (2024) found that intermediate diel thermal variability (2.2°C change per day) yielded the most effective thermal preconditioning for fast‐growing, branching species, while more “hardy” or slow‐growing species were less influenced by thermal preconditioning. In addition to the level of variability, the difference in photosystem response between coral hosts may also be attributed to the differing species of associated algal symbionts hosted (*Symbiodinium* spp. vs. *Breviolum* spp.). To the best of our knowledge, there is currently no published literature that has addressed the response of the endosymbionts *ex‐hospite* to a variable temperature regime, so it is unclear whether species associations would play a role. Investigating ideal temperature treatments for coral‐algal associations would improve standardization and comparisons across heat stress studies (Grottoli et al. 2021).

### 4.2 Suppression of Stress Response Genes in Temperature‐Treated *A. cervicornis* and *P. clivosa*

Several differentially expressed genes identified in our study have been previously implicated in various stages of the coral heat stress response and coral thermal tolerance. First, nitric oxide synthase activity was significantly downregulated in variable temperature‐treated *A. cervicornis* . This enzyme has been previously observed to produce nitric oxide, a reactive oxygen species, in the coral host, and lower levels of nitric oxide have been correlated with greater bleaching resilience (Hawkins et al. 2013, 2014). The overproduction of reactive oxygen species due to thermal stress can trigger the coral immune response, which increases the production of antioxidants to combat this oxidative stress (Helgoe et al. 2024). Another oxidative detoxification gene that was differentially expressed in variable temperature‐treated *A. cervicornis* was cytochrome P450. This gene was one of 55 that were differentially expressed in heat acclimatized *A. hyacinthus* which were reciprocally transplanted to high variability tidal pools in American Samoa (Palumbi et al. 2014). Several oxidases and peroxidases were also downregulated in variable temperature‐treated *A. cervicornis* in our study, which, along with the downregulation of nitric oxide synthase activity and cytochrome P450, suggests that the variable temperature regime did not cause an overproduction of reactive oxygen species even though temperatures reached a sublethal level of stress for several hours each day.

In *Symbiodinium* spp., there was a signal of upregulation in metabolic and antioxidant activity based on gene expression of the variable temperature‐treated *A. cervicornis* . As there was less photophysiological damage compared to controls based on photosynthetic efficiency and coral tissue coloration, the upregulation in metabolic activity may reflect greater productivity in the algal endosymbionts due to the variable temperature regime. However, the upregulation of antioxidant activity could indicate a signal of stress response for the algal endosymbionts, as previous work in *A. aspera* detected significant upregulation of cytochrome and antioxidant genes following exposure to short‐term heat stress (Rosic et al. 2014). After 28 days of exposure to elevated temperatures, cultured *Symbiodinium* spp. also demonstrated differential expression of stress response genes and photosynthetic machinery (Gierz et al. 2017), and so the variable temperature regime applied in this study may have triggered the stress response of the algal endosymbionts. If the algal endosymbionts in variable temperature‐treated *A. cervicornis* experienced stress, then the gene expression patterns of the host would be reactive, yet GO terms related to the heat stress response and immune response were significantly downregulated. This incongruence between host and symbiont may be due to timing, whereby the symbiont was starting to experience thermal stress, but the host response was not triggered. Alternatively, this may be indicative of underlying changes in the algal endosymbiont communities, as previous research has documented the suppression of the host innate immune response during symbiont selection and community maintenance (Jacobovitz et al. 2021).

The differential gene expression patterns observed in *A. cervicornis* following the variable temperature treatment suggest a dampening of the coral ESR, as many stress and immune response genes were significantly downregulated. This is supported by the correlative analysis of GO terms which were categorized into two types of ESRs based on the *Acropora* meta‐analysis, where GO terms in this study were positively correlated with the type “B” ESR (Dixon et al. 2020). Previous correlations with the meta‐analysis found positive relationships with the type “A” ESR following thermal stress exposure (Aichelman et al. 2024; Drury et al. 2022; Wuitchik et al. 2024). Overall, the alignment with the type “B” ESR indicates that the variable temperature regime did not cause significant stress at the cellular level for the *A. cervicornis* host.

While GO analysis could not be conducted on the *P. clivosa* dataset due to the low number of DEGs, the gene annotations for this coral host also support a suppression of the stress response due to significant downregulation of HSP and protein folding activity. A previous study which applied short‐term heat stress to *P. clivosa* found patterns of upregulation of HSPs, collagens, and TNF receptor‐associated factors (Avila‐Magaña et al. 2021), which suggests that the variable temperature regime in this study did not activate the thermal stress response in *P. clivosa*. The lack of DEGs in *Breviolum* spp. also supports a lack of stress response in the algal endosymbionts of variable temperature‐treated *P. clivosa*.

### 4.3 Genes Implicated in Chromatin Accessibility May Mediate Acclimatization in the Long‐Term via Epigenetic Modifications

The differential expression of histone genes and GO terms related to epigenetic regulation highlights a potential mechanism for incorporating signatures of acclimatization as non‐genetic markers. Epigenetic modifications influence the accessibility of genes and are hypothesized to play a role in preconditioning, as epigenetic changes can be environmentally driven and can also be inherited across generations in corals (Hackerott et al. 2021). They also, along with the transcriptional plasticity of an individual, may explain why phenotypic benefits are conferred following successive thermal stress events (Hughes et al. 2019). In this study, the histone H2A gene was significantly upregulated in variable temperature‐treated *A. cervicornis* . Canonical histone 2A is involved in DNA compaction into nucleosomes and thus influences chromatin accessibility for gene expression (Talbert and Henikoff 2010). Post‐translational phosphorylation of a variant of this histone, H2A.X, which is involved in the DNA damage response, has been linked to nutrient and thermal stress in *A. cervicornis* (Rodriguez‐Casariego et al. 2018). However, there were no significant differences in H2A.X gene expression between treatments, which is in agreement with a study on toxin exposure in the Eastern oyster but contradictory to a study in the freshwater hydrozoan, *Hydra* (Gonzalez‐Romero et al. 2017; Reddy et al. 2017; Rodriguez‐Casariego et al. 2018). The disagreement in gene expression of chromatin‐associated proteins and post‐translational histone modifications may again be due to the timing of sampling, and further work on this topic is necessary to understand the molecular mechanisms of epigenetic regulations following an environmental change in corals.

While the upregulation of histone H2A may be one potential mechanism of variable temperature acclimatization, several GO terms in variable temperature‐treated *A. cervicornis* related to epigenetic regulation of gene expression and other histone modifications, namely H3‐K9 trimethylation, were downregulated. As previous work has demonstrated that DNA methylation influences phenotypic acclimatization in corals through the regulation of gene expression (Dixon et al. 2018; Eirin‐Lopez and Putnam 2019; Rodríguez‐Casariego et al. 2020), the differential expression patterns observed in this study may activate or repress gene expression based on the stability of the DNA‐histone interactions either opening or hindering genomic region accessibility (Nawaz et al. 2022). Future work in variable temperature preconditioning should incorporate the analysis of both DNA and histone modifications to determine whether the gene expression patterns observed in this study lead to incorporations at the epigenetic level.

## 5 Conclusion

Overall, this work contributes to the field of thermal preconditioning through the novel characterization of both *A. cervicornis* and *P. clivosa* host and symbiont gene expression response. In *A. cervicornis*, 3°C daily oscillations of sublethal temperature stress led to greater gene regulation and dampening of the ESR, which may signal acclimatization or reallocation of energy expenditure as a result of an environmental change. However, thermal variability differentially impacted algal photophysiology, whereby treated *A. cervicornis* maintained higher photosynthetic efficiencies following short‐term heat stress, while treatment did not influence the performance of *P. clivosa* in heat stress. The dosage applied may not have been sufficient to observe a change in phenotype for *P. clivosa*, a known stress‐tolerant species. Future studies should build upon this research by investigating different durations and dosages of thermal variability across a diversity of reef‐building species. In tandem, expanding genomic resources and increasing coral host and symbiont gene expression datasets across more species and thermal stress exposures will allow for the generation of more meta‐analyses, which will improve the current understanding of the molecular mechanisms of acclimatization.

## Author Contributions

**Allyson DeMerlis:** data curation (lead), formal analysis (lead), methodology (equal), project administration (equal), resources (equal), software (lead), validation (lead), visualization (lead), writing – original draft (lead). **Michael S. Studivan:** data curation (equal), formal analysis (equal), funding acquisition (lead), investigation (equal), resources (equal), software (equal), supervision (equal), validation (equal), visualization (equal), writing – review and editing (equal). **Kevin Wong:** data curation (equal), formal analysis (equal), software (equal), supervision (equal), validation (equal), visualization (equal), writing – review and editing (equal). **Nash Soderberg:** methodology (equal), project administration (equal), resources (equal). **David Ehrens:** investigation (equal), methodology (equal), project administration (equal). **Lys M. Isma:** investigation (equal), methodology (equal), project administration (equal). **Katrina Sophia Cocson:** data curation (equal), formal analysis (equal), methodology (equal), project administration (equal). **Katrina Rosing:** investigation (equal), methodology (equal), project administration (equal). **Rowan Thomas:** investigation (equal), methodology (equal), project administration (equal). **Danielle Dvorkin:** methodology (equal), project administration (equal). **Patrick M. Kiel:** investigation (equal), methodology (equal), project administration (equal), writing – review and editing (equal). **Joseph D. Unsworth:** project administration (equal), resources (equal), writing – review and editing (equal). **Martine D'Alessandro:** project administration (equal), resources (equal). **Ana M. Palacio‐Castro:** validation (equal), writing – review and editing (equal). **Diego Lirman:** project administration (equal), resources (equal), supervision (equal), writing – review and editing (equal). **Andrew C. Baker:** resources (equal), supervision (equal), writing – review and editing (equal). **Erinn M. Muller:** supervision (equal), writing – review and editing (equal). **Nikki Traylor‐Knowles:** conceptualization (equal), formal analysis (equal), investigation (equal), methodology (equal), resources (equal), supervision (equal), writing – review and editing (equal). **Ian C. Enochs:** conceptualization (equal), data curation (equal), formal analysis (equal), funding acquisition (lead), investigation (equal), methodology (lead), project administration (equal), resources (lead), software (equal), supervision (lead), validation (equal), visualization (equal), writing – review and editing (equal).

## Disclosure

*Benefit‐sharing statement*: Benefits from this research were created collaboratively with students and researchers in the United States, between a university, cooperative institute, and federal research laboratory. The results of this study benefit Florida's Coral Reef, which is a priority for environmental conservation. All data and scripts to reproduce results for this study are freely accessible via NCBI, GitHub, and Zenodo.

## Conflicts of Interest

The authors declare no conflicts of interest.

## Supporting information


**Data S1:** ece372108‐sup‐0001‐DataS1.zip.

## Data Availability

All statistical analyses were conducted using R v4.2.1 (R Development Core Team 2011). Analysis scripts and outputs are publicly available on GitHub (DeMerlis 2023b) and is archived on Zenodo (https://doi.org/10.5281/zenodo.17055645). Raw sequence files are available on the National Center for Biotechnology Information (NCBI) Sequence Read Archive (SRA) under BioProject PRJNA1196005. Annotated transcriptomes and associated files are publicly available on GitHub for *A. cervicornis* (Studivan 2023c), *P. clivosa* (Studivan 2023d), *Symbiodinium* spp. (Studivan 2023b), and *Breviolum* spp. (Studivan 2023a).
