# Supplementary material for: Differences in Molecular Responses to a Thermally Variable Preconditioning Treatment for Two Caribbean Coral Species
Source: Ecol Evol. 2025 Nov 5;15(11):e72108. doi: 10.1002/ece3.72108 (PMC12588177; doi:10.1002/ece3.72108)
Supplement: Supplementary file 1 — Data S1: ece372108‐sup‐0001‐DataS1.zip. [file ECE3-15-e72108-s001.zip › ece372108-sup-0001-Supinfo.docx]

Differences in molecular responses to a thermally variable preconditioning treatment for two Caribbean coral species

Allyson DeMerlis^1,2,3^, Michael S. Studivan^1,2^, Kevin Wong^3^, Nash Soderberg^1,2^, David Ehrens^3^, Lys M. Isma^3^, Katrina Rosing^3^, Katrina Sophia Cocson^3^, Rowan Thomas^3^, Danielle Dvorkin^3^, Patrick M. Kiel^1,2^, Joseph D. Unsworth^3^, Martine D’Alessandro^3^, Ana M. Palacio-Castro^1,2^, Diego Lirman^3^, Andrew C. Baker^3^, Erinn M. Muller^4^, Nikki Traylor-Knowles^3^, Ian C. Enochs^2^

1. University of Miami Cooperative Institute for Marine and Atmospheric Studies, Miami, FL, USA
2. U.S. National Oceanic and Atmospheric Administration Atlantic Oceanographic and Meteorological Laboratory, Miami, FL, USA
3. University of Miami Rosenstiel School for Marine, Atmospheric, and Earth Science, Miami, FL, USA
4. Mote Marine Laboratory, Sarasota, FL, USA

#### **Supplementary Information**

#### **Methodology**

#### *Calcification*

To assess impacts of the variable temperature treatment on coral calcification, the wet weights of fragments were obtained following the buoyant weighing technique at the beginning and the end of the treatment (Davies, 1989). A multiparameter digital water quality meter (YSI Pro1030 pH and Conductivity Meter, #6051030) was used to measure the salinity and temperature of the water at the time of each buoyant weight measurement, and these values were used to determine seawater density. Then, coral weight in water was converted to coral weight in air using the following equation (Using density of solid aragonite as 2.93 g cm^-3^): ${mass}_{Air}=\frac{{mass}_{Seawater}}{1 - \frac{{Density}_{Seawater}}{{DensityAragonite}}}$ ​​

Initial and final weights were converted to a percent change and tested for statistical differences based on treatment, with each species assessed separately. Outliers were removed using the *car* R package (Fox, 2024). For *A. cervicornis*, data met normality and variance assumptions for using a one-way ANOVA. For *P. clivosa*, data were not normally distributed and variances were unequal, so a non-parametric Kruskal-Wallis test was used.

#### *Coral Tissue Coloration*

Coral tissue coloration was assessed for each fragment at the beginning and end of the variable temperature treatment using photography (Canon Powershot G1X) with a Kodak Grey Scale and Coral Color Reference Card attached (Siebeck et al., 2006). Photos were captured during peak daytime settings for the aquaria lights (09:00–14:00). Using ImageJ software, photos were converted to a gray scale and then red (R) intensities were extracted for four points on each coral, as this metric correlates with chlorophyll-*a* concentration of the algal endosymbiont communities (Schneider et al., 2012; Winters et al., 2009). A higher R-intensity value (range: 0–255) indicates a color closer to white, i.e. bleaching. The mean of the four R-intensity values measured per coral fragment was taken for downstream analysis. To evaluate changes in R-intensity based on the variable temperature treatment, endpoint measurements were normalized to initial values and were statistically tested using a non-parametric Kruskal-Wallis test, as data were not normally distributed and variances were unequal.

####

#### **Supplementary Figures**


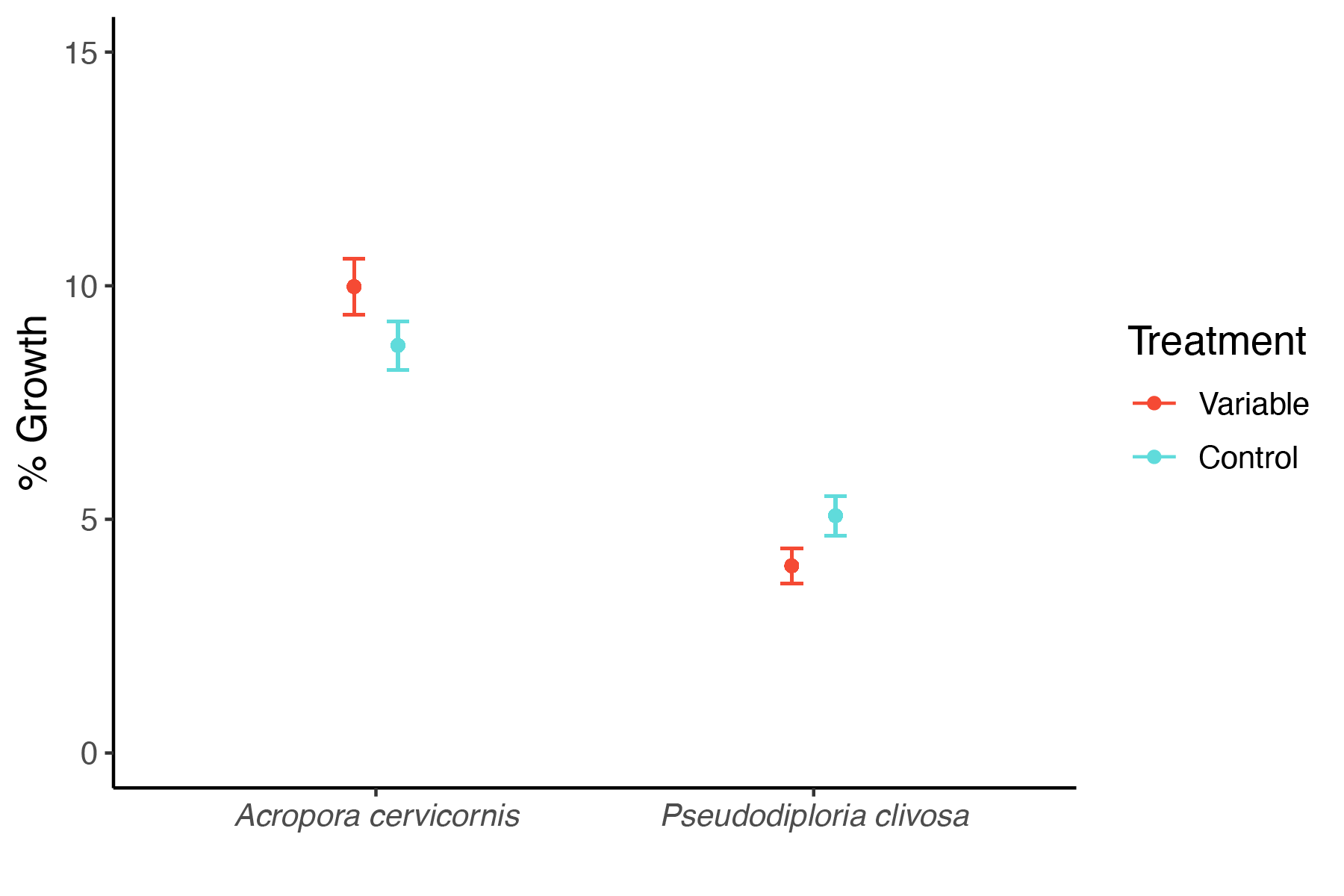


**Figure S1.** Average percent change in calcification following the 28 d variable temperature treatment (red) compared to controls (blue). Error bars represent the standard error of the mean. Statistical tests provided in Table S3.


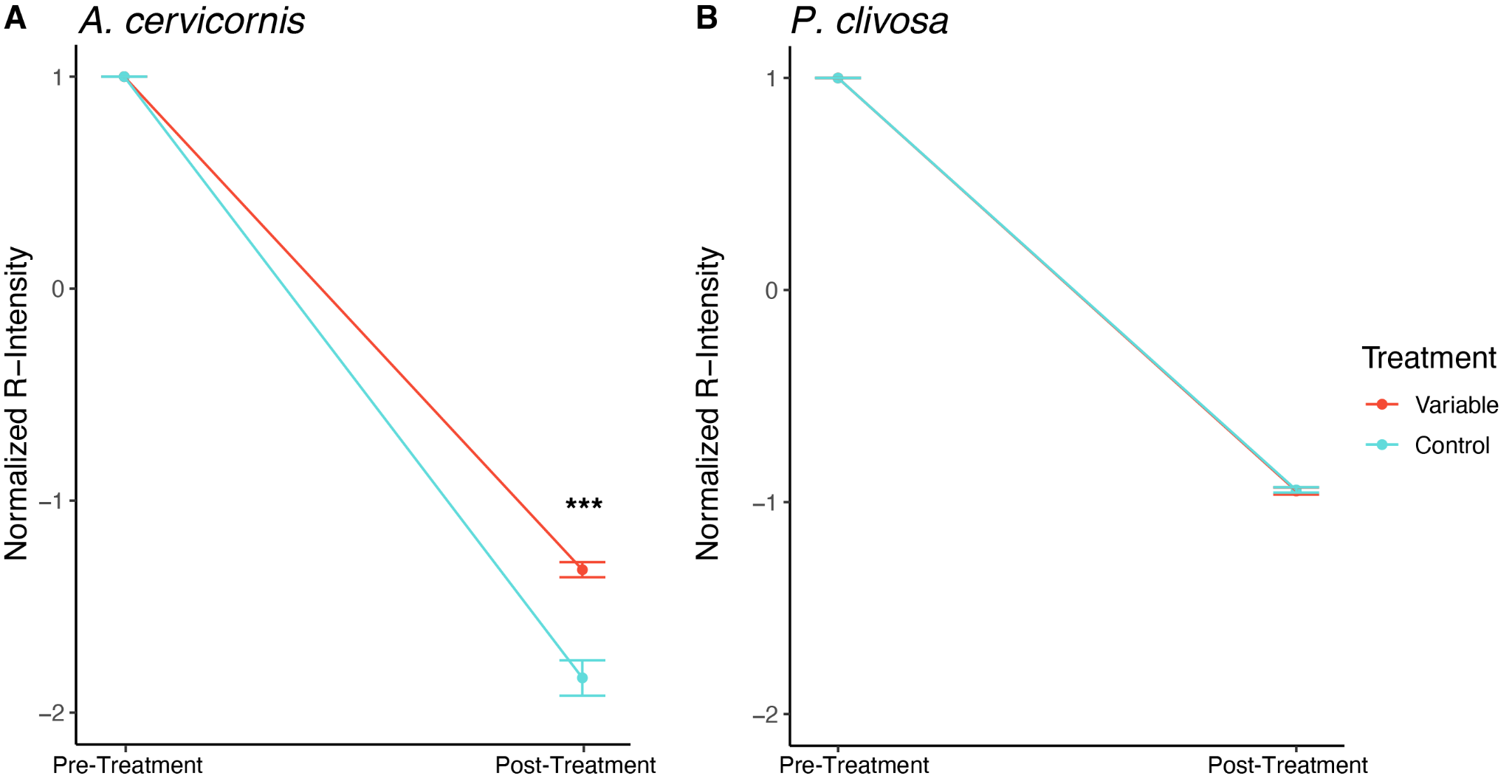


**Figure S2.** Relative change in coral tissue coloration over the treatment period (measured as R-intensity) normalized to initial measurement for **A)** *A. cervicornis* and **B)** *P. clivosa.* The average R-intensity for each treatment is plotted with error bars representing the standard error of the mean. Final values were multiplied by -1 to visualize the reduction in tissue coloration. The significant p-value following a Kruskal-Wallis test is denoted with asterisk, significance code ^(^***^)^ p<0.001 (Table S4).

####
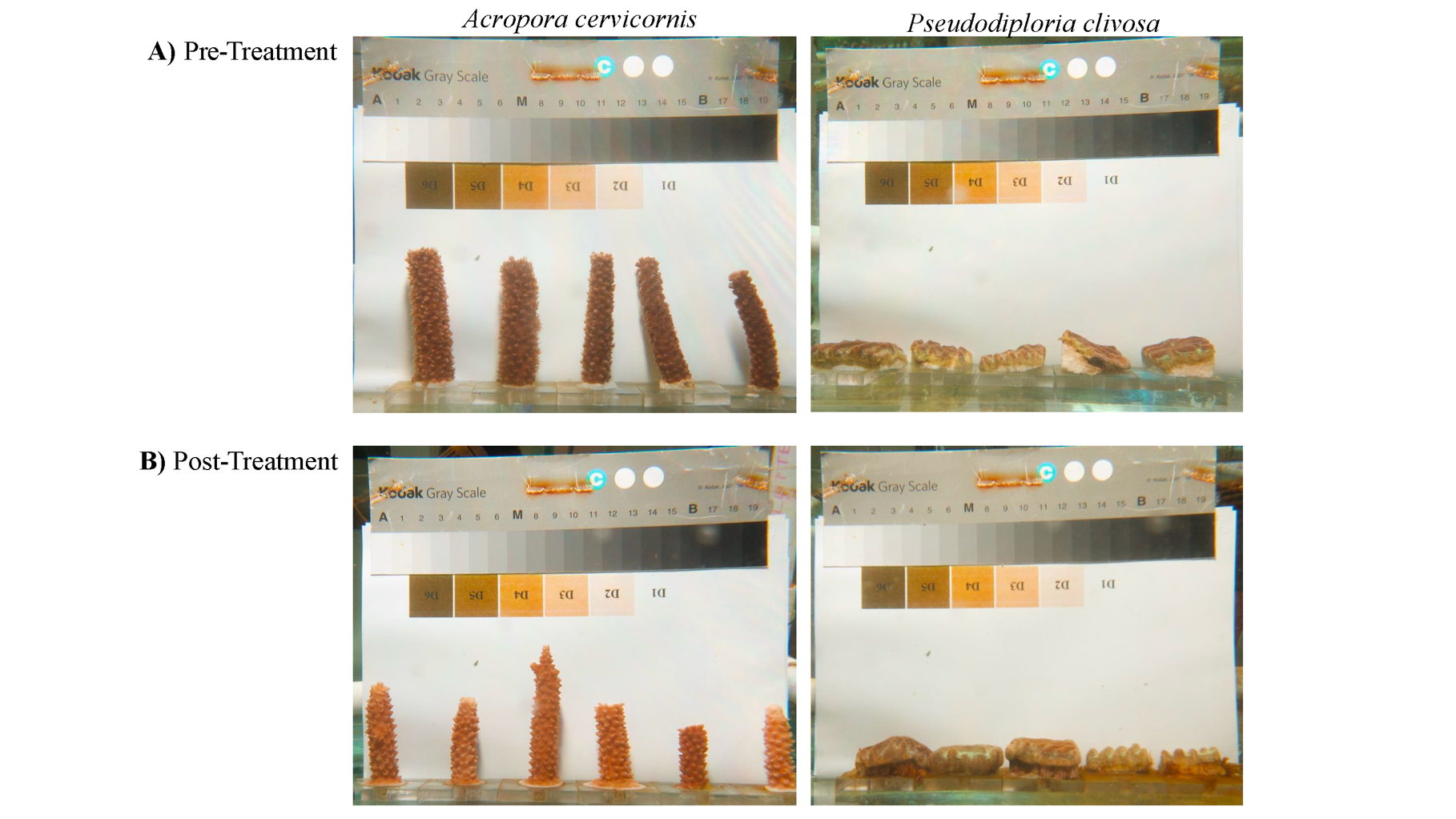


**Figure S3.** Representative photos of *A. cervicornis* and *P. clivosa* fragments from the beginning and end of the 28-day variable temperature treatment. No corals displayed signs of paling or bleaching during the variable temperature treatment.

####

#### **Supplementary Tables**

**Table S1.** Number of fragments per colony and species at the start of the experiment.

| **Species** | **Colony** | **StagDB MLG Clonal ID (Genotype)** | **Number of Fragments** | **Total** |
| --- | --- | --- | --- | --- |
| *A. cervicornis* | BC-8B | HG0960 | 50 | 146 |
|  | MB-B | HG0965 | 51 |  |
|  | SI-C | HG0944 | 45 |  |
| *P. clivosa* | A | N/A | 49 | 149 |
|  | B | N/A | 51 |  |
|  | C | N/A | 49 |  |

**Table S2.** Tank conditions throughout the experiment.

| **Measurement** | **Value** | **Unit** | **Equipment** |
| --- | --- | --- | --- |
| Tank turnover | 250–300 | mL min-1 | Nanostream 6040, Tunze |
| Light conditions  Photosynthetically active radiation (PAR) | 250–300 (peak illumination) | μmol m−2 s−1 | PAR meter: MQ-200, Apogee |
| Light cycle | Diurnal, off from 19:00–6:00, followed by a 3-h increase, a 7-h hold, and a 3-h decrease |  | Lights: Hydra 52 HD, Aqua Illumination |
| Feeding (broadcast feeding method, once per night) | 5.5 | μg mL−1 | Food: Reef-Roids, Polyplab |

**Table S3.** Statistical tests of percent change in calcification following variable temperature treatment.

| **Species** | **Effect** | **Test** | **N** | **df** | **Test Statistic** | **Pr(>F)** |
| --- | --- | --- | --- | --- | --- | --- |
| *Acropora cervicornis* | Variable Treatment | ANOVA | 53 | 1 | 2.526 | 0.118 |
| *Pseudodiploria clivosa* | Variable Treatment | Kruskal-Wallis | 67 | 1 | 3.216 | 0.0729 |

**Table S4.** Kruskal-Wallis test of normalized R-intensity following variable temperature treatment.

| **Species** | **Effect** | **N** | **df** | **H** | **Pr(>F)** | **Signif.** |
| --- | --- | --- | --- | --- | --- | --- |
| *Acropora cervicornis* | Variable Treatment | 133 | 1 | 19.067 | 1.26E-05 | *** |
| *Pseudodiploria clivosa* | Variable Treatment | 142 | 1 | 0.434 | 0.51 |  |

**Table S5.** Welch’s ANOVA of normalized photosynthetic efficiency following variable temperature treatment.

| **Species** | **Effect** | **df1** | **df2** | **F** | **Pr(>F)** | **Signif.** |
| --- | --- | --- | --- | --- | --- | --- |
| *Acropora cervicornis* | Variable Treatment | 1 | 115.99 | 101.22 | <2.2E-16 | *** |
| *Pseudodiploria clivosa* | Variable Treatment | 1 | 95.163 | 16.709 | 9.10E-05 | *** |

**Table S6.** One-way ANOVAs to evaluate treatment differences of the photosynthetic efficiency of *A. cervicornis* at each rapid heat-stress assay temperature.

| **Effect** | **Rapid Heat-Stress Assay Temperature** | **df** | **SS** | **MS** | **F** | **Pr(>F)** | **Signif.** |
| --- | --- | --- | --- | --- | --- | --- | --- |
| Variable Treatment | 28 | 1 | 1.0760E-02 | 1.0760E-02 | 5.587 | 0.032 | * |
| Residuals | 28 | 15 | 2.8890E-02 | 1.9260E-03 |  |  |  |
| Variable Treatment | 30 | 1 | 5.2350E-03 | 5.2350E-03 | 3.365 | 0.090 |  |
| Residuals | 30 | 13 | 2.0227E-02 | 1.5560E-03 |  |  |  |
| Variable Treatment | 32 | 1 | 5.5870E-03 | 5.5870E-03 | 3.208 | 0.094 |  |
| Residuals | 32 | 15 | 2.6119E-02 | 1.7410E-03 |  |  |  |
| Variable Treatment | 33 | 1 | 2.2720E-02 | 2.2716E-02 | 10.23 | 0.006 | ** |
| Residuals | 33 | 15 | 3.3310E-02 | 2.2210E-03 |  |  |  |
| Variable Treatment | 34 | 1 | 6.3360E-03 | 6.3360E-03 | 4.7 | 0.049 | * |
| Residuals | 34 | 13 | 1.7524E-02 | 1.3480E-03 |  |  |  |
| Variable Treatment | 35 | 1 | 1.6870E-02 | 1.6869E-02 | 7.129 | 0.018 | * |
| Residuals | 35 | 15 | 3.5490E-02 | 2.3660E-03 |  |  |  |
| Variable Treatment | 36 | 1 | 1.5610E-02 | 1.5606E-02 | 6.157 | 0.025 | * |
| Residuals | 36 | 16 | 4.0560E-02 | 2.5350E-03 |  |  |  |
| Variable Treatment | 37 | 1 | 3.6000E-04 | 3.6100E-04 | 0.147 | 0.707 |  |
| Residuals | 37 | 14 | 3.4280E-02 | 2.4490E-03 |  |  |  |

**Table S7.** One-way ANOVAs to evaluate treatment differences of the photosynthetic efficiency of *P. clivosa* at each rapid heat-stress assay temperature.

| **Effect** | **Rapid Heat-Stress Assay Temperature** | **df** | **SS** | **MS** | **F** | **Pr(>F)** | **Signif.** |
| --- | --- | --- | --- | --- | --- | --- | --- |
| Variable Treatment | 28 | 1 | 1.9200E-03 | 1.9200E-03 | 0.975 | 0.339 |  |
| Residuals | 28 | 15 | 2.9540E-02 | 1.9690E-03 |  |  |  |
| Variable Treatment | 30 | 1 | 3.3700E-03 | 3.3700E-03 | 2.265 | 0.150 |  |
| Residuals | 30 | 18 | 2.6780E-02 | 1.4880E-03 |  |  |  |
| Variable Treatment | 32 | 1 | 2.4030E-03 | 2.4030E-03 | 1.643 | 0.218 |  |
| Residuals | 32 | 16 | 2.3400E-02 | 1.4630E-03 |  |  |  |
| Variable Treatment | 33 | 1 | 2.9130E-03 | 2.9130E-03 | 2.041 | 0.172 |  |
| Residuals | 33 | 16 | 2.2836E-02 | 1.4270E-03 |  |  |  |
| Variable Treatment | 34 | 1 | 1.9000E-04 | 1.8600E-04 | 0.071 | 0.793 |  |
| Residuals | 34 | 17 | 4.4340E-02 | 2.6080E-03 |  |  |  |
| Variable Treatment | 35 | 1 | 1.0600E-04 | 1.0650E-04 | 0.061 | 0.809 |  |
| Residuals | 35 | 15 | 2.6364E-02 | 1.7576E-03 |  |  |  |
| Variable Treatment | 36 | 1 | 3.3350E-03 | 3.3350E-03 | 1.693 | 0.214 |  |
| Residuals | 36 | 14 | 2.7573E-02 | 1.9690E-03 |  |  |  |
| Variable Treatment | 37 | 1 | 1.4930E-03 | 1.4930E-03 | 1.271 | 0.275 |  |
| Residuals | 37 | 17 | 1.9972E-02 | 1.1750E-03 |  |  |  |

​​

**Table S8.** Test statistics for PERMANOVA results of differential gene expression analysis for each host species and associated symbiont.

| **Species** | **Factor** | **df** | **Test statistic** | **R^2^** | **p** |
| --- | --- | --- | --- | --- | --- |
| *Acropora cervicornis* | Colony | 2 | 5.587 | 0.182 | 1.00E-06 |
|  | Treatment | 2 | 5.580 | 0.182 | 1.00E-06 |
| *Symbiodinium* spp. | Colony | 2 | 1.588 | 0.065 | 2.48E-04 |
|  | Treatment | 2 | 2.495 | 0.101 | 1.00E-06 |
| *Pseudodiploria clivosa* | Colony | 2 | 2.622 | 0.110 | 1.00E-06 |
|  | Treatment | 2 | 1.736 | 0.073 | 1.11E-03 |
| *Breviolum spp.* | Colony | 2 | 0.991 | 0.055 | 0.413 |
|  | Treatment | 2 | 1.111 | 0.061 | 0.149 |

**​​Table S9.** Counts of significant differentially expressed genes for each host species and associated symbiont, filtered based on a criteria of p-adj < 0.05 and |L2FC| > 1.

| **Species** | **Comparison** | **Up-Regulated DEGs** | **Down-Regulated DEGs** | **Total DEGs** |
| --- | --- | --- | --- | --- |
| *Acropora cervicornis* | Variable vs. Control | 200 | 207 | 407 |
|  | Variable vs. Initial | 489 | 565 | 1054 |
|  | Control vs. Initial | 1050 | 943 | 1993 |
| *Symbiodinium* spp. | Variable vs. Control | 0 | 2 | 2 |
|  | Variable vs. Initial | 50 | 21 | 71 |
|  | Control vs. Initial | 28 | 13 | 41 |
| *Pseudodiploria clivosa* | Variable vs. Control | 3 | 7 | 10 |
|  | Variable vs. Initial | 33 | 31 | 64 |
|  | Control vs. Initial | 38 | 23 | 61 |
| *Breviolum spp.* | Variable vs. Control | 0 | 0 | 0 |
|  | Variable vs. Initial | 1 | 0 | 0 |
|  | Control vs. Initial | 0 | 0 | 0 |

**Table S10.** Number of significant gene ontology (GO) terms based on log-2-fold change (L2FC) and log-transformed p-value (lpv) for each species and contrast. GO terms are separated into three categories: Biological Process (BP), Molecular Function (MF), and Cellular Component (CC).

|  |  | **GO Term (L2FC)** | | | **GO Term (lpv)** | | |
| --- | --- | --- | --- | --- | --- | --- | --- |
| **Species** | **Contrast** | **BP** | **MF** | **CC** | **BP** | **MF** | **CC** |
| *Acropora cervicornis* | Variable vs. Initial | 802 | 117 | 156 | 869 | 151 | 185 |
|  | Control vs. Initial | 651 | 105 | 149 | 684 | 129 | 176 |
|  | Variable vs. Control | 160 | 15 | 89 | 218 | 24 | 100 |
| *Pseudodiploria clivosa* | Variable vs. Initial | 0 | 0 | 8 | 7 | 7 | 25 |
|  | Control vs. Initial | 0 | 0 | 0 | 0 | 0 | 0 |
|  | Variable vs. Control | 0 | 0 | 0 | 0 | 0 | 0 |

**Table S11.** List of *A. cervicornis* DEGs for DESeq2 contrasts containing the variable temperature-treated corals with gene annotations.

(See “TableS11_Acervicornis_variable_DEGs_annotated.xlsx” attached file)

**​​Table S12.** List of *Symbiodinium* DEGs for DESeq2 contrasts containing the variable temperature-treated corals with gene annotations.

(See “TableS12_Symbiodinium_variable_DEGs_annotated.xlsx” attached file)

**​​Table S13.** List of *P. clivosa* DEGs for DESeq2 contrasts containing the variable temperature-treated corals with gene annotations.

(See “TableS13_Pclivosa_variable_DEGs_annotated.xlsx” attached file)

**​​Table S14.** List of *Breviolum* DEGs for DESeq2 contrasts containing the variable temperature-treated corals with gene annotations.

(See “TableS14_Breviolum_variable_DEGs_annotated.xlsx” attached file)

**​​**

**Table S15.** List of significantly enriched GO terms (BP, MF, and CC) in variable temperature-treated *A. cervicornis*.

(See “TableS15_Acervicornis_sigGOterms_uniquevariable.xlsx” attached file)

**Table S16.** List of significantly enriched GO terms (BP only) in variable temperature-treated *A. cervicornis* which were shared with the Dixon et al. (2020) *Acropora* meta-analysis “Type A” response.

(See “TableS16_Acervicornis_TypeA_GOterms_VariablevsDixon.xlsx” attached file)

**Table S17.** List of significantly enriched GO terms (BP only) in variable temperature-treated *A. cervicornis* which were shared with the Dixon et al. (2020) *Acropora* meta-analysis “Type B” response.

(See “TableS17_Acervicornis_TypeB_GOterms_VariablevsDixon.xlsx” attached file)

####

####

#### **References**

[Davies, P. S. (1989). Short-term growth measurements of corals using an accurate buoyant weighing technique. *Marine Biology*, *101*](http://paperpile.com/b/BrkzYW/2jxh), 389–395. https://doi.org/[10.1007/BF00428135](http://dx.doi.org/10.1007/BF00428135)

Dixon, G., Abbott, E., & Matz, M. (2020). Meta-analysis of the coral environmental stress response: *Acropora* corals show opposing responses depending on stress intensity. *Molecular Ecology*, *29*(15), 2855–2870.

[Fox, J. (2024). *car: Companion to Applied Regression*.](http://paperpile.com/b/BrkzYW/kppI) https://cran.r-project.org/web/packages/car/index.html

Schneider, C. A., Rasband, W. S., & Eliceiri, K. W. (2012). NIH Image to ImageJ: 25 years of image analysis. [*Nature Methods*, *9*(7), 671–675. https://doi.org/](http://paperpile.com/b/BrkzYW/zC1sv)[10.1038/nmeth.2089](http://dx.doi.org/10.1038/nmeth.2089)

[Siebeck, U. E., Marshall, N. J., Klüter, A., & Hoegh-Guldberg, O. (2006). Monitoring coral bleaching using a colour reference card. *Coral Reefs* , *25*](http://paperpile.com/b/BrkzYW/fVKxX)(3), 453–460.

Winters, G., Holzman, R., Blekhman, A., Beer, S., & Loya, Y. (2009). Photographic assessment of coral chlorophyll contents: Implications for ecophysiological studies and coral monitoring. [*Journal of Experimental Marine Biology and Ecology*](http://paperpile.com/b/BrkzYW/jhuzs), [*380*(1-2), 25–35. https://doi.org/](http://paperpile.com/b/BrkzYW/jhuzs)[10.1016/j.jembe.2009.09.004](http://dx.doi.org/10.1016/j.jembe.2009.09.004)
